# β-Lapachone Ameliorates Lipotoxic Cardiomyopathy in Acyl CoA Synthase Transgenic Mice

**DOI:** 10.1371/journal.pone.0091039

**Published:** 2014-03-10

**Authors:** Moon Hee Jeong, Nguyen Khoi Song Tran, Tae Hwan Kwak, Byung Keon Park, Chul Soon Lee, Tae-Sik Park, Young-Hoon Lee, Woo Jin Park, Dong Kwon Yang

**Affiliations:** 1 College of Life Sciences, Gwangju Institute of Science and Technology, Gwangju, Korea; 2 Department of Life Science, Gachon University, Sungnam, Korea; 3 R&D Center, KT&G Life Sciences Corp., Suwon, Korea; 4 Department of Oral Anatomy, School of Dentistry and Institute of Oral Biosciences, Chonbuk National University, Jeonju, Korea; University of Western Ontario, Canada

## Abstract

Lipotoxic cardiomyopathy is caused by myocardial lipid accumulation and often occurs in patients with diabetes and obesity. This study investigated the effects of β-lapachone (β-lap), a natural compound that activates Sirt1 through elevation of the intracellular NAD^+^ level, on acyl CoA synthase (ACS) transgenic (Tg) mice, which have lipotoxic cardiomyopathy. Oral administration of β-lap to ACS Tg mice significantly attenuated heart failure and inhibited myocardial accumulation of triacylglycerol. Electron microscopy and measurement of mitochondrial complex II protein and mitochondrial DNA revealed that administration of β-lap restored mitochondrial integrity and biogenesis in ACS Tg hearts. Accordingly, β-lap administration significantly increased the expression of genes associated with mitochondrial biogenesis and fatty acid metabolism that were down-regulated in ACS Tg hearts. β-lap also restored the activities of Sirt1 and AMP-activated protein kinase (AMPK), the two key regulators of metabolism, which were suppressed in ACS Tg hearts. In H9C2 cells, β-lap-mediated elevation of AMPK activity was retarded when the level of Sirt1 was reduced by transfection of siRNA against Sirt1. Taken together, these results indicate that β-lap exerts cardioprotective effects against cardiac lipotoxicity through the activation of Sirt1 and AMPK. β-lap may be a novel therapeutic agent for the treatment of lipotoxic cardiomyopathy.

## Introduction

Myocardial metabolic abnormalities, which occur in response to various factors including obesity and diabetes, are significant risk factors for heart failure [1], [2]. The imbalance between caloric intake and expenditure is related to obesity and metabolic disorders. When caloric intake exceeds caloric expenditure, excess calories are normally stored in adipocytes in the form of triacylglycerol (TG). Once storage capacity of TG in adipocyte is exceeded, it begins to accumulate in non-adipose tissues, including the heart [3], [4]. In addition, the fuel used in myocardial metabolism switches from fatty acids (FAs) to glucose in pathological conditions including cardiac hypertrophy, ischemia, myocardial infarction, and heart failure [5]–[7]. Lipids accumulate abnormally when this metabolic switch, which is thought to be an adaptive response, occurs frequently in the heart. This accumulation can be cytotoxic and cause cardiac dysfunction that ultimately leads to lipotoxic cardiomyopathy [8], [9].

Sirt1, which belongs to the yeast Silent information regulator (Sir2) family, is a member of the NAD^+^-dependent class III group of histone deacetylases (HDACs). Recent studies indicated that Sirt1 plays a pivotal role in a variety of cellular processes including gene silencing, DNA damage repair, and apoptosis/cell survival, and extends life span [10], [11]. Sirt1 is also a critical regulator of metabolic processes including lipolysis, FA oxidation, mitochondrial biogenesis, and gluconeogenesis [12]–[14]. Sirt1 was recently shown to repress the onset of diet-induced obesity by promoting mitochondrial FA oxidation through activating peroxisome proliferator-activated receptor α (PPARαα and its coactivator PGC-1α [15], [16]. Notably, resveratrol, a Sirt1 activator, protects against obesity and type 2 diabetes in mice fed with a high-fat diet [12], [17].

β-lapachone (β-lap), a natural *o*-naphthoquinone compound, is a substrate of NADH:quinone oxidoreductase (NQO1). NQO1 mediates the reduction of β-lap by using NADH as an electron donor [18]. Reduced β-lap is unstable and rapidly re-oxidized. This futile β-lap redox cycle is coupled with oxidation of NADH to NAD^+^. As Sirt1 activity strictly requires NAD^+^ as a cofactor, β-lap is thought to increase Sirt1 activity by increasing the cytoplasmic NAD^+^/NADH ratio [19], [20].

Since Sirt1 protects against metabolic disorders by facilitating FA oxidation, we hypothesized that β-lap-mediated activation of Sirt1 activity can prevent lipotoxic cardiomyopathy. To test this hypothesis, we investigated the effects of β-lap administration in transgenic (Tg) mice overexpressing acyl CoA synthase (ACS), which have severe lipotoxic cardiomyopathy due to excess import of FAs into cardiomyocytes [21].

In this study, we show that oral administration of β-lap to ACS Tg mice reduced lipid accumulation in the heart and attenuated lipotoxicity-induced heart failure through increasing Sirt1 and AMPK activities.

## Materials and Methods

### Animal models

All animal experiments in this study were performed with the approval of the Animal Care Committee of Gwangju Institute of Science and Technology. αMHC-ACS-transgenic mice were kindly provided by Dr. Jean E. Schaffer (Washington University, St. Louis, MO, USA). Mice were used and maintained at room temperature on a 12 hrs light:dark schedule. Mice were anesthetized by intraperitoneal injection with a mixture of ketamine (100 mg/kg) and xylazine (5 mg/kg). For euthanasia, mice were injected with the same amount of ketamine (100 mg/kg) and xylazine (5 mg/kg) mixture, and hearts were rapidly excised.

### β-lapachone administration

β-lapachone (β-lap; 3,4-dihydro-2,2-dimethyl-2H-naphthol[1,2b]pyran-5,6-dione) was chemically synthesized by the R&D Center, KT&G Life Sciences (Suwon, Korea). The compound was freshly dissolved in vehicle solution (5 mg/ml sodium lauryl sulphate), and daily administered by oral gavage with a dose of 50 mg/kg/day as previously described [19], [20].

### Histological analysis of heart sections

Mice were sacrificed and hearts were arrested at the end diastole. Paraffin or glycol methacrylate-embedded (Technovit 8100, Kultzer&Co) hearts were cut into 4-µm and 1.5-µm thick slices, respectively. The sections were stained with hematoxylin-eosin solution. To measure the surface area of cardiomyocytes, suitable cross sections with nearly circular capillary profiles and nuclei were selected. Approximately, 400 cells per each heart were observed under an Axiophot microscope (Carl Zeiss) and analyzed using AnalySIS 2.3 software (Carl Zeiss). Fibrotic areas in the heart sections were measured after trichrome staining. The degree of fibrosis was calculated as percentage of the fibrotic area in relation to the total heart area. These sections were observed as described for hematoxylin-eosin stained sections.

### Quantitative real-time PCR (qRT-PCR)

Hearts were removed, weighed, and snap-frozen in liquid nitrogen. Total RNA was isolated using TRI reagent (Sigma). Reverse-transcriptase reactions were performed using ImProm II reverse-transcriptase (Promega) with oligo-dT priming. qRT-PCR was performed using a TaKaRa Thermal Cycler Dice Real Time System Single TP815 (Takara Bio) and SYBR Green (Takara Bio) as the fluorescent dye. The sequences of the PCR primers used in this study are listed in Table S1.

### Echocardiography

Mice were anesthetized and the chests were shaved. Echocardiography was performed using a Powervision 6000 instrument with a 12-MHz microprobe (Toshiba). Hearts were scanned using M-mode guided by a short-axis view of the 2-dimensional mode. Frozen frames were printed using a video graphic printer (Sony).

### Measurement of TG in heart tissue

Mice were euthanized by CO_2_ inhalation and hearts were isolated. Heart tissue (60 mg) was homogenized in phosphate-buffered saline (PBS). Heart extracts were mixed with 1 ml of chloroform:methanol (2∶1) and vortexed for 1 min, centrifuged for 10 min, and the organic phase was then collected and dried under nitrogen gas. These dried lipid extracts were solubilized in 500 µl chloroform containing 1% Triton-X100 and dried under nitrogen gas. The dried lipids were solubilized in TG assay buffer and the TG content was measured enzymatically using the Triglyceride Quantification Kit (Abcam) according to the manufacturer's instruction. Colorimetric changes were measured at 570 nm using a multimode reader (Tecan Group Ltd).

### Electron microscopy

Heart slices were fixed with 3% glutaraldehyde in PBS for 3 h. Fixed samples were washed five times with 0.1 M cacodylate buffer (pH 7.2) containing 0.1% CaCl_2_ at 4°C and post-fixed in 0.1 M cacodylate buffer containing 0.1% CaCl_2_ and 1% OsO_4_ for 2 h at 4°C. After rinsing with cold distilled water, samples were slowly dehydrated with an ethanol series and propylene oxide at 4°C, and then embedded in Spurr's epoxy resin [22]. After polymerization of the resin at 70°C for 36 h, serial sections were cut with a diamond knife on an ultramicrotome (Leica, Buffalo Grove) and mounted on formvar-coated slot grids. Sections were stained with 4% uranyl acetate for 10 min and lead citrate for 7 min, and were observed using an H-7650 transmission electron microscope (Hitachi).

### Western blotting

Heart extracts were lysed in RIPA buffer (1% NP-40, 50 mM Tris-HCl [pH 7.4], 150 mM NaCl, and 10 mM NaF) with protease inhibitor cocktail (Roche Diagnostics) and a phosphatase inhibitor cocktail (Sigma). Protein homogenates were separated on an SDS-PAGE gel and transferred to a PVDF membrane (Bio-Rad Laboratories). After blocking with 5% non-fat milk for 1 h, the membrane was incubated overnight at 4°C with antibodies against Complex 2 (Mito Sciences), LKB1 (Abcam), FOXO-1αPARP-1, ACSL1, p-AMPK, AMPK, p-ACC, ACC, GAPDH (all from Cell Signaling) or p53 (Santa Cruz Biotechnology). The membrane was subsequently incubated with HRP-conjugated secondary antibodies (AbFrontier) and developed using a chemiluminescent substrate (PerkinElmer).

### Real-time PCR of mitochondrial DNA (mtDNA)

MtDNA and nuclear DNA were isolated from heart tissue using a DNeasy kit (Qiagen). The relative quantity of mtDNA and nuclear DNA was assessed by qRT-PCR. The sequences of primers used to amplify the mitochondrial genes, cytochrome c oxidase subunit 1 (mt-Co1), cytochrome b (mt-Cyt b), and the nuclear gene H19 are listed in Table S1.

### Immunoprecipitation

Heart tissues (1 mg) or cells (500 µg) were lysed in RIPA buffer containing a protease inhibitor cocktail, 10 µM trichostatin A, and 10 mM nicotinamide. Equal amounts of extracts were incubated with anti-acetyl-lysine agarose beads (ImmuneChem) on a rotation wheel overnight at 4°C. The resulting protein complex was washed four times with a RIPA buffer and separated by SDS-PAGE, followed by immunoblotting with specific primary antibodies.

### Cell culture and shRNA transfection

H9C2 cells were obtained from the Korean Cell Line Bank, and were grown in Dulbecco's modified Eagle's medium (Hyclone) supplemented with 10% fetal bovine serum (Hyclone) and antibiotics (Invitrogen). Sirt1-shRNA and scramble-shRNA were synthesized by GeneScript and subcloned into the RNAi-Ready pSIREN-DNR-DsRed-Express vector (Clontech). The resulting vectors were transfected into H9C2 cells using Lipofectamin 2000 (Invitrogen) according to the manufacturer's instructions. After incubation for 24 h, H9C2 cells were treated with 5 µM β-lap for 30 min as previously described [19].

### Statistics

All data are reported as mean ± SD. Statistical significance was analyzed using the Student's *t* test or two-way ANOVA for multiple comparisions (Statview 5.0, SAS). *p*<.05, or *p*<0.001 was considered statistically significant.

## Results

### β-lap attenuates adverse cardiac remodeling in ACS Tg mice

**Figure 1:**
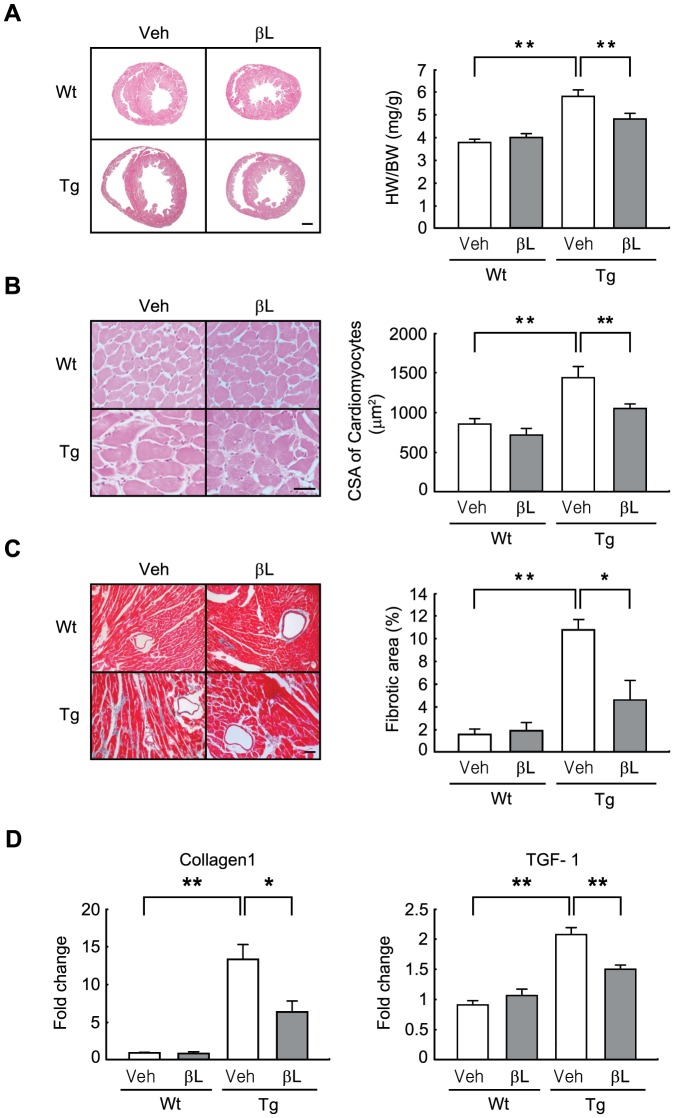
β-Lap attenuates cardiac hypertrophy and fibrosis in ACS Tg mice. **A**. Cross-sections of the hearts and assessment of heart weight/body weight (HW/BW) ratios in Wt and ACS Tg mice administered either vehicle (Veh) or β-lap (βL). Scale bar is 1 mm. **B**. Higher magnification images of the heart sections. Cell surface area (CSA) of individual cardiomyocytes was measured using the AnalySIS image analysis program. Scale bar is 50 µm. **C**. Trichrome staining of histological sections. Fibrotic areas were quantified using the AnalySIS image analyzer on histological sections. Scale bar is 100 µm. **D**. qRT-PCR analysis of collagen I and TGF- β1 transcript levels in mouse hearts. n = 4−6 per group. Significance was measured via two-way ANOVA. *p<0.05, **p<0.001.

Ten-week old wild-type (Wt) and ACS Tg mice were administered vehicle or β-lap daily for 6 weeks. Heart weight to body weight (HW/BW) ratio was 52% higher in vehicle-administered ACS Tg mice than in vehicle-administered Wt mice. However, the HW/BW ratio was only 36% higher in β-lap-administered ACS Tg mice than in β-lap-administered Wt mice (Fig. 1A). Microscopic analysis of histological sections revealed that the cross-sectional areas (CSA) of cardiomyocytes was 67% larger in vehicle-administered ACS Tg mice than in vehicle-administered Wt mice, and this increase was significantly inhibited by β-lap-administration (41% increase vs. β-lap-administered Wt mice) (Fig. 1B).

Heart failure is associated with increased interstitial fibrosis. Heart sections were subjected to trichrome staining, and then the fibrotic area was measured. The fibrotic areas were significantly increased in vehicle-administered ACS Tg mice (6.6-fold increase vs. vehicle-treated Wt mice); however, this increase was significantly inhibited by β-lap administration (2.9-fold increase vs. β-lap-administered Wt mice) (Fig. 1C). qRT-PCR further indicated that expression levels of collagen 1 and TGF- β1, which are markers of fibrosis, were significantly lower in β-lap-administered ACS Tg mice than in vehicle-administered ACS Tg mice (Collagen 1, 52% decrease; TGF- β1, 27% decrease vs. vehicle-administered ACS Tg mice) (Fig. 1D).

Collectively, these data indicate that β-lap treatment offers protection to the development of adverse cardiac remodeling in ACS Tg mice.

### β-lap ameliorates cardiac dysfunctions in ACS Tg mice

**Figure 2:**
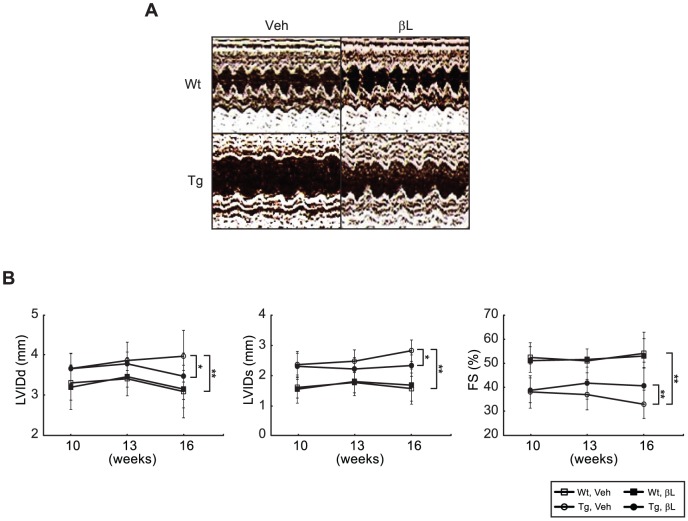
β-Lap preserves cardiac functions in ACS Tg mice. **A**. Two-dimensional guided M-mode echocardiographic images were obtained from Wt and ACS Tg mice administered either vehicle (Veh) or β-lap (βL). **B**. Quantification of cardiac structure and function assessed by echocardiography. The leftventricular internal dimension at diastole (LVIDd), leftventricular internal dimension at systole (LVIDs), and fractional shortening (FS) values are shown. n = 9−12 per group. Significance was measured via two-way ANOVA. *p<0.05, **p<0.001.

Echocardiography was performed to evaluate cardiac functions in ACS Tg mice. When 10 weeks old, which was the age at which β-lap administration began, ACS Tg mice exhibited significant left ventricular dilation compared to Wt mice (4% increase in left ventricular internal dimension at diastole (LVIDd) and 32% increase in left ventricular internal dimension at systole (LVIDs) vs. Wt mice). These structural alterations were associated with reduced contractile function in ACS Tg mice (27% decrease in fractional shortening (FS) vs. Wt mice). After administration of β-lap for six weeks, while this contractile dysfunction worsened over time in vehicle-administered ACS Tg mice, β-lap-administered ACS Tg mice showed the reduction of left ventricular dilation and contractile dysfunction compared to Veh-treated Tg mice (10% decrease in LVIDd, 17% decrease in LVIDs and 23% increase in FS vs. Veh-treated Tg mice) (Figs. 2A and B). The detailed echocardiography parameters are summarized in Table 1. These data indicate that β-lap ameliorates cardiac dysfunction in ACS Tg mice.

**Table 1:** Echocardiographic parameters.

	Wt, Veh	Wt, βL	Tg, Veh	Tg, βL
HR, bpm	472±12	483±17	462±25	468±32
IVSTd, mm	0.52±0.05	0.56±0.04	0.56±0.03	0.56±0.04
LVIDd, mm	2.98±0.35	3.18±0.20	4.32±0.14**	3.86±0.15≠
LVPWd, mm	0.65±0.04	0.62±0.09	0.61±0.04	0.64±0.03
IVSTs, mm	1.15±0.11	1.36±0.09	1±0.04	1.17±0.02
LVIDs, mm	1.48±0.23	1.46±0.12	3±0.12**	2.31±0.14≠
LVPWs, mm	1.02±0.07	1.2±0.08	0.9±0.04	1.04±0.06
EF	87.67±2.04	89±1.79	63.67±1.83**	77.57±1.81≠ ≠ ≠
FS, %	51.67±2.47	53.6±2.42	29.89±1.17**	40.57±1.81≠ ≠

Wt, wild-type mice; Tg, ACS overexpressing transgenic mice; Veh, vehicle; βL, β-lapachone; IVSTd, Interventricular septum in diastole; LVIDd, Left ventricular internal dimension in diastole; LVPWd, Left ventricular posterior wall thickness in diastole; IVSTs, Interventricular septum in systole; LVIDs, Left ventricular internal dimension in systole; LVPWs, Left ventricular posterior wall thickness in systole; EF, Ejection fraction; FS, Fractional shortening. Significance was measured via two-way ANOVA.

**p<0.001 vs Wt,Veh.

≠ *p*<0.05 vs Tg,Veh.

≠ ≠ *p*<0.001 vs Tg,Veh.

### β-lap diminishes TG accumulations in the heart of ACS Tg mice

**Figure 3:**
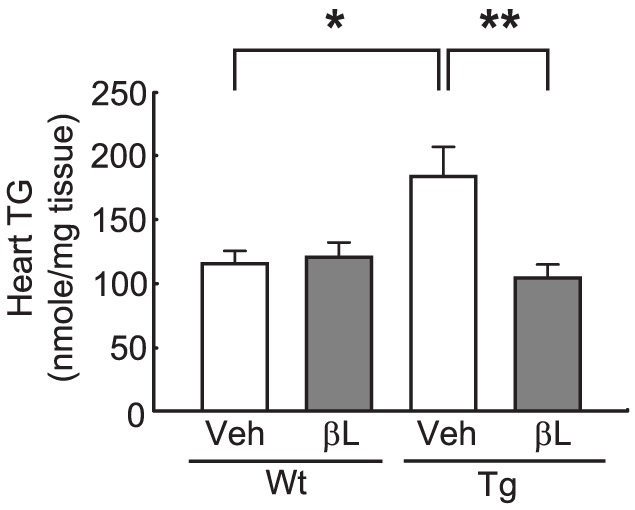
β-Lap prevents lipid accumulation in the heart of ACS Tg mice. Triglyceride contents in hearts from Wt and ACS Tg mice administered either vehicle (Veh) or β-lap (βL). n = 7−8 per group. Significance was measured via two-way ANOVA. *p<0.05, **p<0.001.

Intramyocardial TG accumulation is a hallmark of lipotoxic cardiomyopathy. The TG content in the heart was 36% higher in vehicle-administered ACS Tg mice than in vehicle-administered Wt mice. However, the TG content in the heart was indistinguisheable between β-lap-administered ACS Tg mice and Wt mice (Fig. 3).

### β-lap preserves mitochondrial integrity in ACS Tg mice hearts

**Figure 4:**
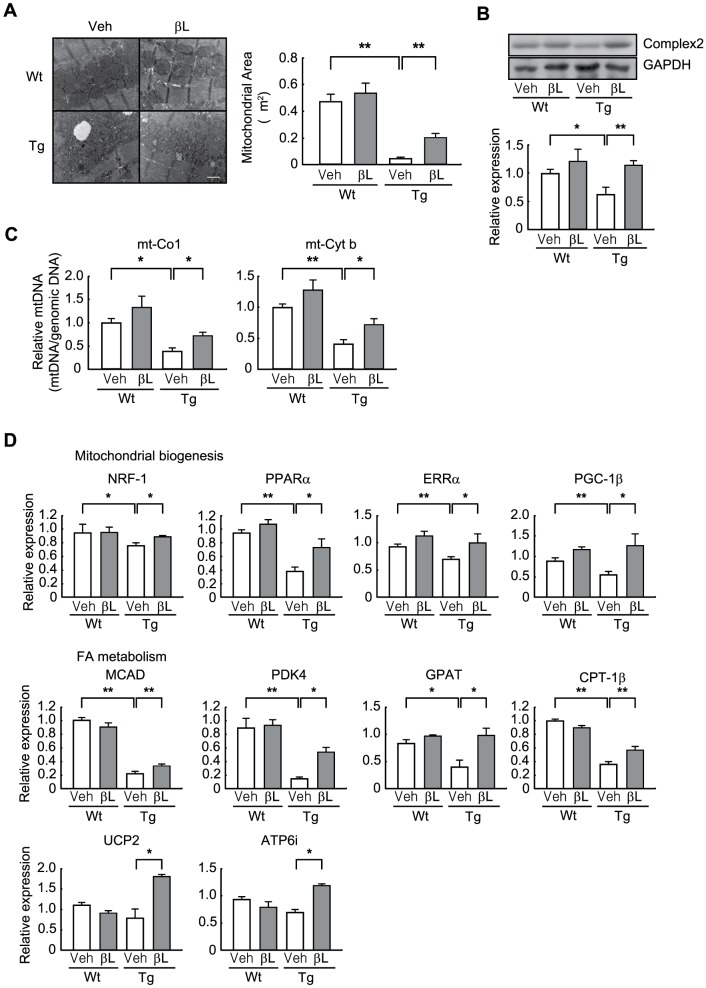
β-Lap preserves mitochondrial structure and functions in ACS Tg mice. **A**. Electron microscopy images of mitochondrial structure and quantification of the mitochondrial surface area in sections of hearts from Wt and ACS Tg mice administered either vehicle (Veh) or β-lap (βL). Scale bar is 1 µm. **B**. Western blot analysis of Complex II protein level. Mice heart lysates (50 µg) were subjected to western blot analysis, and band density was quantified using NIH Image J software. GAPDH was used as a loading control. **C**. qRT-PCR analysis of mt-Co1 and mt-Cyt b transcript levels in mouse hearts. **D**. qRT-PCR analysis of genes involved in mitochondrial biogenesis (NRF-1, PPARα, ERRαα and PGC-1β) and FA metabolism (MCAD, PDK4, GPAT, CPT1-β, UCP2, and ATP6i). n = 4−6 per group. Significance was measured via two-way ANOVA. *p<0.05, **p<0.001.

Many studies have shown that mitochondrial biogenesis and function are impaired in lipotoxic cardiomyopathy associated with obesity and diabetes [23], and that mitochondrial dysfunction is linked to cardiac dysfunction [24]. Therefore, we tested whether β-lap affects mitochondrial integrity and activity in the heart of ACS Tg mice. Electron microscopy revealed that the mitochondrial area was greatly reduced in the hearts of 16-week-old vehicle-administered ACS Tg mice (9.6 fold decrease vs. vehicle-administered Wt mice). However, this decrease was significantly lessened by β-lap treatment (4.2 fold increase in β-lap-administered ACS Tg mice vs. vehicle-administered Tg mice) (Fig. 4A). Similarly, the level of mitochondrial complex II protein was reduced in the hearts of ACS Tg mice, and this was restored by administration of β-lap (Fig. 4B). In addition, β-lap treatment significantly prevented the reduction in the level of mtDNA in the heart of ACS Tg mice (Fig. 4C). The β-lap-mediated restoration of mitochondrial integrity was further confirmed by determining the expression levels of several genes involved in mitochondrial biogenesis including nuclear respiratory factor-1 (NRF-1), peroxisome proliferator-activated receptor α (PPARα), estrogen-related receptor α (ERRα), and peroxisome proliferator-activated receptor γ coactivator-1β (PGC-1β). In addition, genes involved in FA metabolism including medium chain acyl-CoA dehydrogenase (MCAD), pyruvate dehydrogenase kinase 4 (PDK4), glycerol-3-phophate acetyltransferase (GPAT), carnitine palmitoyltransferase 1-β (CPT1-β), uncoupling protein 2 (UCP2) and ATPase, H^+^ transporting [vacuolar proton pump] member 1 (ATP6i), were also up-regulated in β-lap administered ACS Tg mice (Fig. 4D).

### β-lap activates AMPK in a Sirt1-dependent manner

**Figure 5:**
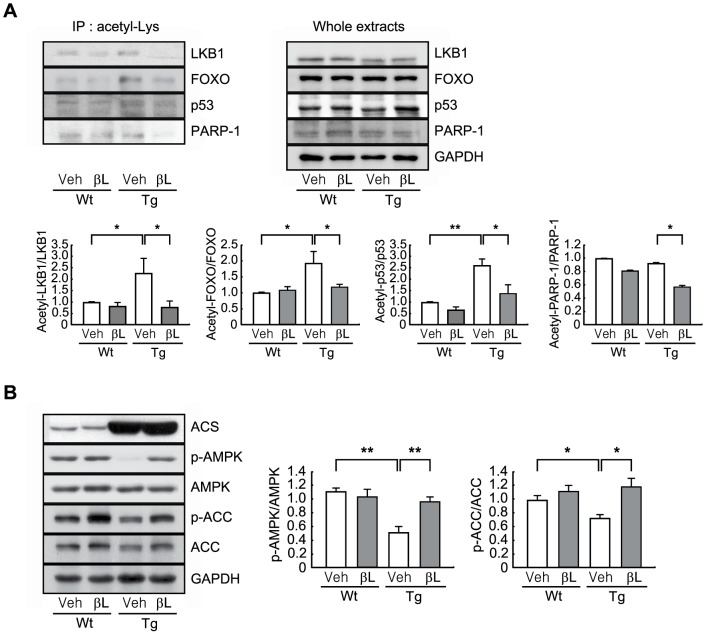
β-Lap stimulates the AMPK signaling pathway through activation of Sirt1. **A**. Mouse hearts lysates (1 mg) were immunoprecipitated with an anti-acetyl lysine antibody. The precipitates and whole extracts (50 µg) were analyzed by western blotting. The levels of the acetylated forms of LKB1, FOXO, p53, and PARP1 were estimated by measuring band densities using NIH Image J software. GAPDH was used as a loading control. **B**. Western blot analysis of the protein levels of ACS, p-AMPK, AMPK, p-ACC, and ACC. Mouse heart lysates (50 µg) were subjected to western blot analysis, and levels of phosphorylated AMPK and ACC were estimated by measuring band densities using NIH Image J software. GAPDH was used as a loading control. n = 3−4 per group. Significance was measured via two-way ANOVA. *p<0.05, **p<0.001.

Sirt1 and AMPK are two key sensors of cellular metabolic status and activation of these molecules corrects a variety of metabolic disorders [25]. We recently showed that β-lap activates Sirt1 through elevating the intracellular NAD^+^ levels [26]. Acetylated proteins were immunoprecipitated from the hearts of Wt or ACS Tg mice using an anti-acetyl-lysine antibody, and then immunoblotted with antibodies against known Sirt1 substrates including LKB1 [27], FOXO [28], p53 [29], and PARP-1 [30]. Acetylation of LKB1, FOXO, p53 was found to be elevated approximately 2-fold in ACS Tg mice, and this was dramatically decreased by the administration of β-lap. ACS Tg mice had similar levels of PARP-1 acetylation as Wt mice, except when treated with β-lap and a 40% decrease was observed in ACS Tg mice (Fig. 5A).

AMPK activity was significantly reduced in the heart of ACS Tg mice, as shown by the reduced phosphorylation of AMPK and one of its downstream targets, acetyl-CoA carboxylase (ACC), and this was completely restored by administration of β-lap (Fig. 5B).

**Figure 6:**
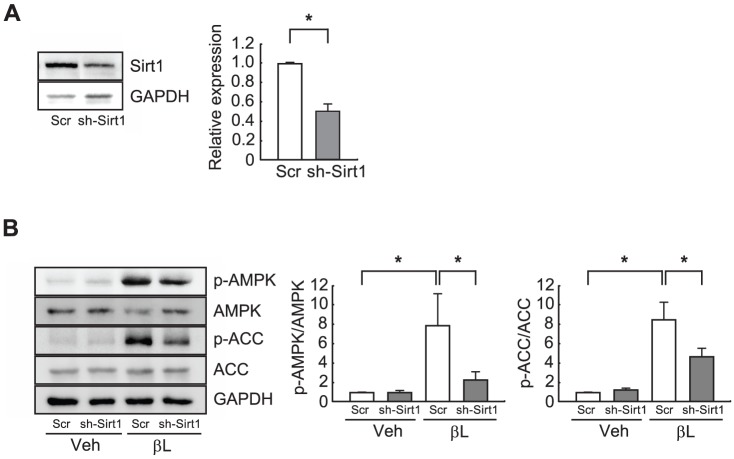
Knockdown of Sirt1 inhibits activation of the AMPK signaling pathway by β-Lap. **A**. H9C2 cells were transfected with a plasmid harboring a scrambled shRNA (Scr) or Sirt1 shRNA (sh-Sirt1). Cell extracts (50 µg) were used for western blot analysis of the Sirt1 protein level and band intensities were quantified using NIH Image J software. GAPDH was used as a loading control. **B**. H9C2 cells transfected with Scr or sh-Sirt1 were treated with vehicle (Veh) or β-lap (βL). Cell extracts (50 µg) were used for western blot analysis of the level of phosphorylated AMPK and ACC and band intensities were quantified using NIH Image J software. GAPDH was used as a loading control. n = 3−4 per group. Significance was measured via Student's t-test (panel A) and two-way ANOVA (panel B). *p<0.05, **p<0.001.

Sirt1 was recently shown to activate AMPK through deacetylating LKB1, a key upstream activator of AMPK [25]. β-lap treatment activated AMPK in cultured H9C2 cells. This β-lap-mediated activation of AMPK was significantly blocked when the level of Sirt1 was reduced by transfection of a si-RNA against Sirt1, sh-Sirt1 (Fig. 6). These data indicate that administration of β-lap may inhibit lipotoxic cardiomyopathy in ACS Tg mice through activation of AMPK in a Sirt1-dependent manner.

## Discussion

In association with diabetes and obesity, TGs and lipid intermediates accumulate in non-adipose tissues, including heart, liver, and muscle. Ectopic lipid overload in the heart leads to lipotoxic cardiomyopathy, which contributes to the fibrosis and dilation of ventricles, and contractile dysfunction [31], [32].

Recently, Sirt1 has been shown that it has a critical role for beneficial effects on mitochondrial function through AMPK activation in many metabolic disease animal models. In mice with diet-induced obesity, treatment with resveratrol, which is an activator of Sirt1, significantly increases mitochondrial biogenesis and protects against metabolic disorders [17]. Resveratrol treatment improves mitochondrial function and increases AMPK activation in mice overexpressing Sirt1, whereas knockout of Sirt1 has none of these benefits [25]. We reasoned that activation of Sirt1 could be an efficient strategy for treatment of lipotoxic cardiomyopathy.

β-lap is a quinone-containing natural compound that is obtained from the bark of the South American Lapacho tree (*Tabebuia avellandedae*) [33]. This compound is an anti-tumor agent with strong cytotoxic activity against a variety of cancer cell lines [34], [35]. A recent study showed that oral administration of β-lap prevents obesity and obesity-related metabolic phenotypes in mice [19], while another study demonstrated that β-lap prevents arterial restenosis in rats by activating AMPK [20]. The pharmacological activity of β-lap is dependent on a FAD-containing enzyme NADH:quinone oxidoreductase (NQO1). NQO1 mediates reduction of β-lap using NADH as an electron source [18]. Thus, β-lap treatment promotes oxidation of NADH to NAD^+^ resulting in the increased intracellular NAD^+^ level. Since Sirt1 activity strictly requires NAD^+^ as a cofactor, β-lap was expected to increase Sirt1 activity. We recently found that this is indeed the case. We demonstrated that β-lap reduces polyQ aggregation and cellular toxicity by inducing autophagy through activation of Sirt1 in a Hungtington's disease model [26].

In the present study, we sought to test the pharmacological role of β-lap against lipotoxic cardiomyopathy in an ACS Tg mouse model. We observed that oral administration of β-lap inhibited heart failure in these mice, accompanied by a reduction of fibrosis and an improvement in heart function (Figs 1 and 2). Furthermore, cardiac lipid accumulation caused by excess import of FAs into the hearts of these Tg mice was diminished by β-lap treatment (Fig 3). Many studies have demonstrated that the lipotoxicity can elevate reactive oxygen species (ROS) production and mitochondrial dysfunction in the heart, and alter cardiac energy metabolism. Diabetic patients have defects in mitochondrial morphology including a reduction in the mitochondrial surface area and an increased number of damaged mitochondria [36], [37]. The heart of ACS Tg mice exhibited similar mitochondrial abnormalities. We found that β-lap improved the morphology of damaged mitochondria in these Tg mice and promoted mitochondrial biogenesis (Fig. 4). Sirt1 and AMPK activities were found to be reduced in the heart of ACS Tg mice, which was reversed by β-lap treatment. Therefore, we suggested that the beneficial effects of β-lap are mediated by the elevated activities of Sirt1 and/or AMPK (Figs 5 and 6).

Collectively, this study shows that pharmacological activation of Sirt1 and/or AMPK using β-lap is a potential strategy for the treatment of lipotoxic cardiomyopathy.

## Supporting Information

Table S1 **Primer sequences used for quantitative real-time PCR.**
(DOCX)
