# Supplementary material for: β-Lapachone Ameliorates Lipotoxic Cardiomyopathy in Acyl CoA Synthase Transgenic Mice
Source: PLoS One. 2014 Mar 10;9(3):e91039. doi: 10.1371/journal.pone.0091039 (PMC3948739; doi:10.1371/journal.pone.0091039)
Supplement: Table S1 — Primer sequences used for quantitative real-time PCR. (DOCX) [file pone.0091039.s001.docx]

**Table S1. Primer sequences used for quantitative real-time PCR**

| Gene | Forward (5’-3’) | Reverse (5’-3’) |
| --- | --- | --- |
| Collagen1 | CGAAGGCAACAGTCGCTTCA | GGTCTTGGTGGTTTTGTATTCGAT |
| TGF-β1 | GTGTGGAGCAACATGTGGAACTCTA | TTGGTTCAGCCACTGCCGTA |
| mt-Co1 | CCCAATCTCTACCAGCATC | GGCTCATAGTATAGCTGGAG |
| mt-Cyt b | TTCTGAGGTGCCACAGTTATT | GAAGGAAAGGTATTAGGGCTAAA |
| H19 | GTACCCACCTGTCGTCC | GTCCACGAGACCAATGACTG |
| NRF1 | TGGAGGAAGCCACCTTACAA | ATGCTCACAGGGATCTGGAC |
| PPARα | TATTCGGCTGAAGCTGGTAC | CTGGCATTTGTTCCGGTTCT |
| ERRα | GGCGACTGCAAGCTCTTC | CAGCCTCAGCATCTTCAATGT |
| PGC1β | CGCTCCAGGAGACTGAATCCAG | CTTGACTACTGTCTGTGAGGC |
| MCAD | ACTGACGCCGTTCAGATTTT | GCTTAGTTACACGAGGGTGATG |
| PDK4 | CGCTTAGTGAACACTCCTTCG | CTTCTGGGCTCTTCTCATGG |
| GPAT | ATCTTCAGAACAGCAAAATCGAAA | CAGCGGAAAACTCCAAATCC |
| CPT1-β | TGCCTTTACATCGTCTCCAA | GGCTCCAGGGTTCAGAAAGT |
| UCP2 | AAAGGGACCTCTCCCAATGT | GGTCGTCTGTCATGAGGTTG |
| ATP6i | CGAACCACCTGAGCTTTCTC | CAAAGAGACCCAGCAGGAAG |
| GAPDH | CTCATGACCACAGTCCATGC | TTCAGCTCTGGGATGACCTT |
